# PinX1 serves as a potential prognostic indicator for clear cell renal cell carcinoma and inhibits its invasion and metastasis by suppressing MMP-2 via NF-κB-dependent transcription

**DOI:** 10.18632/oncotarget.4011

**Published:** 2015-05-27

**Authors:** Hai-Long Li, Li Han, Hai-Rong Chen, Fei Meng, Qing-Hua Liu, Zhen-Qiang Pan, Jin Bai, Jun-Nian Zheng

**Affiliations:** ^1^ The First Clinical Medical College, Nanjing Medical University, Nanjing, Jiangsu, China; ^2^ Jiangsu Key Laboratory of Biological Cancer Therapy, Xuzhou Medical College, Xuzhou, Jiangsu, China; ^3^ Department of Clinical Oncology, The Affiliated Hospital of Xuzhou Medical College, Xuzhou, Jiangsu, China; ^4^ Jiangsu Center for the Collaboration and Innovation of Cancer Biotherapy, Cancer Institute, Xuzhou Medical College, Xuzhou, Jiangsu, China; ^5^ Department of Oncological Sciences, Icahn School of Medicine at Mount Sinai, New York, NY, USA; ^6^ Department of Occupational Medicine and Environmental Health, School of Public Health, Nanjing Medical University, Nanjing, Jiangsu, China

**Keywords:** pinX1, metastasis, prognostic, MMP-2, NF-κB

## Abstract

PIN2/TRF1-interacting telomerase inhibitor 1 (PinX1) is a novel cloned gene which has been identified as a major haploinsufficient tumor suppressor essential for maintaining telomerase activity, the length of telomerase and chromosome stability. This study explored the clinical significance and biological function of PinX1 in human clear cell renal cell carcinoma (ccRCC). The clinical relevance of PinX1 in ccRCC was evaluated using tissue microarray and immunohistochemical staining in two independent human ccRCC cohorts. Our data demonstrated that PinX1 expression was dramatically decreased in ccRCC tissues compared with normal renal tissues and paired adjacent non-tumor tissues. Low PinX1 expression was significantly correlated with depth of invasion, lymph node metastasis and advanced TNM stage in patients, as well as with worse overall and disease-specific survival. Cox regression analysis revealed that PinX1 expression was an independent prognostic factor for ccRCC patients. Moreover, PinX1 inhibited the migration and invasion of ccRCC by suppressing MMP-2 expression and activity via NF-κB-dependent transcription *in vitro*. *In vivo* studies confirmed that PinX1 negatively regulated ccRCC metastasis and the expression of MMP-2 and NF-κB-p65. These findings indicate that PinX1 suppresses ccRCC metastasis and may serve as a ccRCC candidate clinical prognostic marker and a potential therapeutic target.

## INTRODUCTION

Kidney cancer is estimated to have been diagnosed about 63, 920 new cases in 2014 in the United States [1]. Most kidney tumors are renal cell carcinomas (RCC), and 70% are the clear cell type (ccRCC) [2]. Although there has been a steady decline in the cancer death rate over the past 2 decades (it depends on the prevention, early detection, and treatment) [1], but once ccRCC happens metastatic, it remains largely incurable [3], median survival of the patient is only about 13 months [4]. However, the molecular mechanism contributes to regulating the invasion and metastasis of ccRCC remains unclear. Any insight into the mechanisms of ccRCC metastasis may contribute to the development of more effective and specific strategies to interfere with ccRCC progression. Thus, novel diagnosis, prognosis and individualized medication biomarkers are strongly needed for ccRCC.

Human telomeres are DNA-protein complexes which cap and protect the ends of linear chromosomes and are essential for elongating telomeres and maintaining chromosome stability [5, 6]. Telomerase has been confirmed that it can be activated in most human cancers [7, 8]. Telomerase contains a catalytic subunit at its core which is called human telomerase reverse transcriptase (hTERT). hTERT is the rate-limiting enzyme of telomerase and is well known to be activated by deregulation of many oncogenes and tumor suppressors [9, 10]. In addition, the ability of telomerase to elongate telomeres is regulated by telomere-associated proteins [11], including telomeric repeat binding factor 1 (TRF1) [12] and its associated proteins [13, 14], including PinX1 [15]. However, unlike other TRF1-binding proteins, PinX1 is unique in that it can also directly bind to hTERT and inhibit telomerase activity [15].

PinX1 is a novel cloned gene which consists of seven exons in humans and localizes at human chromosome 8p23, a region frequently associated with loss of heterozygosity in a variety of human malignancies [16–18]. It has been identified as a major haploinsufficient tumor suppressor essential for maintaining telomerase activity, the length of telomerase and chromosome stability [15, 19, 20]. Zhang et al. reported that ectopic overexpression or suppression of PinX1 leads to a decrease or an increase in both telomerase activity and cancer cell tumorigenicity [19]. Moreover, the role of PinX1 as a putative tumor suppressor was proved by several other groups in different cancer cell lines, such as human breast cancer cells, hepatoma cells, burkitt's lymphoma cells and esophageal epithelial cells [20–23]. On the other hand, Ma et al. reported that LOH of PinX1 played major role in gastric carcinoma development, which suggested PinX1 might has a potential inhibitory role in cancer metastasis [24]. Then, increasing evidence demonstrated that PinX1 plays a key role in cancer progression [25–27]. However, PinX1 expression status and its correlation with the clinicopathological features in ccRCC have never been investigated. In addition, the potential molecular mechanisms underlying the role of PinX1 in ccRCC are still unknown.

In this study, we investigated the clinicopathological and prognostic significance as well as the potential role of PinX1 in the development and progression of ccRCC. Our data demonstrated that loss of PinX1 expression was significantly associated with ccRCC progression. Meanwhile, we demonstrated that PinX1 suppresses ccRCC invasion and metastasis by inhibiting the expression and activity of MMP-2 via NF-κB-dependent transcription *in vitro* and *in vivo*. These data imply that PinX1 may be used as a potential prognosis and therapeutic marker for this aggressive ccRCC.

## RESULTS

### PinX1 expression is decreased in human ccRCC

**Figure 1:**
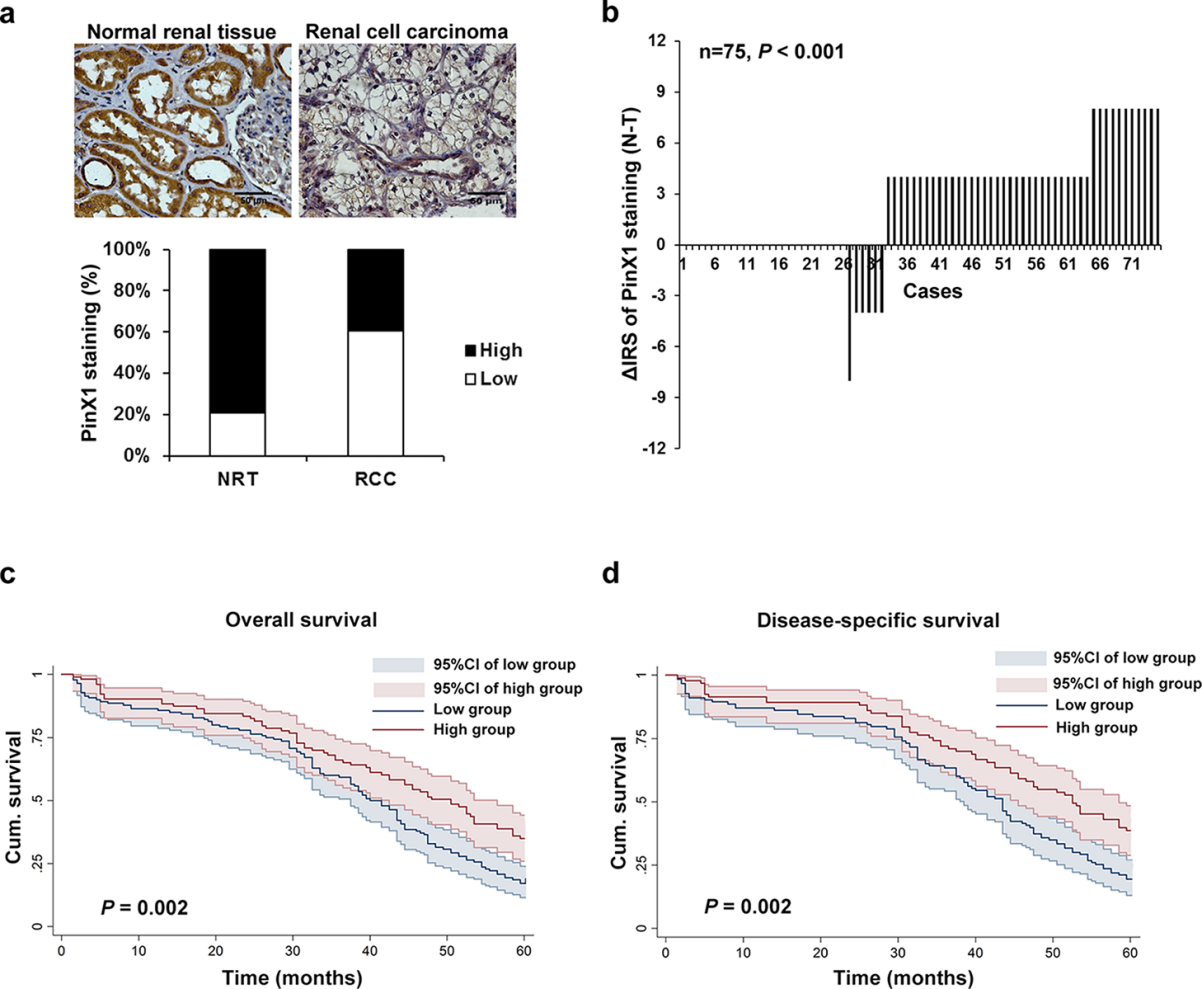
Expression of PinX1 is decreased in ccRCC tissues and associated with 5-year overall and disease-specific survival in ccRCC patients **a.** top panel, representative photographs taken at 400 magnification showed PinX1 immunohistochemical staining in normal renal tissue and clear cell renal cell carcinoma. Bottom panel, PinX1 expression staining was lower in ccRCC tissues than in normal renal tissues. Immunohistochemical staining data was available from 35 normal renal tissues and 278 ccRCC tissues. **b.** The distribution of the difference in PinX1 staining (ΔIRS = IRS_N_–IRS_T_). Immunoreactivity score (IRS) of PinX1 staining was available from 75 pairs of tissues; *P* values were calculated with the Wilcoxon test. PinX1 expression was lower in tumor tissues (T) compared with paired adjacent non-tumor tissues (N). **c.** Low PinX1 expression correlated with a poorer 5-year overall cumulative survival for 243 ccRCC patients (*P* = 0.002, log-rank test). **d.** Low PinX1 expression correlated with a poorer 5-year disease-specific cumulative survival for 216 ccRCC patients (*P* = 0.002, log-rank test). Cum. indicates cumulative.

In order to determine whether PinX1 expression is changed in human ccRCC. Immunohistochemistry staining was utilized in TMA slides to evaluate the PinX1 expression in normal renal tissues, clear cell renal cell carcinoma tissues and paired adjacent non-tumor tissues. Samples with IRS 0–3 and IRS 4–12 were classified as low and high expression of PinX1. In validation cohort TMA which contains 278 cases ccRCC tissues and 35 cases normal renal tissues (Figure 1a, top panel), PinX1 low expression staining was observed in 7 of 35 (20%) normal renal tissues, and 168 of 278 (60%) ccRCC tissues (*P* < 0.001, Figure 1a, bottom panel). In training cohort TMA slide containing 75 cases ccRCC tissues with paired adjacent non-tumor tissues, we observed that a significantly lower expression of PinX1 in tumor tissues compared with paired adjacent non-tumor tissues (*P* < 0.001, Figure 1b). Taken together, PinX1 expression is decreased in ccRCC tissues compared with paired adjacent non-tumor tissues and normal renal tissues.

### Decreased PinX1 expression correlates with clinicopathological parameters in ccRCC patients

The clinicopathologic characteristics of the training cohort and the validation cohort of ccRCC biopsies were summarized in Table 1. As shown in Table 1, two sided Fisher's exact analysis revealed that PinX1 expression in the carcinoma tissues of the training cohort conspicuously correlated with some clinicopathological features, such as depth of invasion-pT status (*P* = 0.018), lymph node metastasis-pN status (*P* = 0.043), and TNM stage (*P* = 0.013). These findings were confirmed in the validation cohort of ccRCC patients (Table 1). However, we did not find significant correlation between PinX1 expression with other clinicopathologic features in both training cohort and validation cohort, including age, gender and tumor size.

**Table 1:** Relationship between PinX1 staining and clinicopathological characteristics of the individuals in two cohorts of ccRCC patients

Variables	Training cohort (75 cases)			Validation cohort (278 cases)		
	Low (%)	High (%)	P*	Low (%)	High (%)	P*
**Age**						
≤56 years	19 (52.8)	17 (47.2)	0.216	83 (61.9)	51 (38.1)	0.355
>56 years	16 (41.0)	23 (59.0)		85 (59.0)	59 (41.0)	
**Gender**						
Male	26 (52.0)	24 (48.0)	0.449	111 (58.7)	78 (41.3)	0.238
Female	9 (36.0)	16 (64.0)		57 (64.0)	32 (36.0)	
**Tumor size**						
≤7 cm	10 (47.6)	11 (52.4)	0.560	132 (61.4)	83 (38.6)	0.321
>7 cm	25 (46.3)	29 (53.7)		36 (57.1)	27 (42.9)	
**pT status**						
pT_1_–pT_2_	26 (41.9)	36 (58.1)	0.018	125 (55.8)	99 (44.2)	0.001
pT_3_–pT_4_	11 (84.7)	2 (15.3)		43 (79.6)	11 (20.4)	
**pN status**						
pN_0_	31 (43.7)	40 (56.3)	0.043	143 (57.7)	105 (42.3)	0.006
pN_1_–pN_3_	4 (100.0)	0 (0.0)		25 (83.3)	5 (16.7)	
**TNM stage**						
I–II	22 (38.6)	35 (61.4)	0.013	124 (56.1)	97 (43.9)	0.002
III–IV	13 (72.2)	5 (27.8)		44 (77.2)	13 (22.8)	

* Two sided Fisher's exact tests.

### PinX1 serves as a potential independent molecular prognostic indicator for ccRCC

To further study whether reduced PinX1 staining in ccRCC patients correlates with a worse prognosis, Kaplan-Meier survival curves were constructed using 5-year overall or disease-specific cumulative survival to compare the patients with high PinX1 staining to those with low PinX1 staining (*n* = 243, follow-up time, 60 months). Our data revealed that low PinX1 staining correlated with both worse overall and disease-specific survival in ccRCC (*P* = 0.002 and *P* = 0.002, respectively, log-rank test; Figure 1c and 1d). The 5-year overall cumulative survival rate dropped from 35.0% in patients with high PinX1 expression to 17.1% in those with low PinX1 expression, and the 5-year disease-specific cumulative survival rate dropped from 38.7% in patients with high PinX1 expression to 19.5% in those with low PinX1 expression.

Moreover, we examined whether PinX1 expression was an independent prognostic factor for ccRCC. We performed a univariate Cox regression analysis including PinX1 expression, age, tumor size, pT status, pN status, and TNM stage to study the effects of PinX1 on patients’ survival in ccRCC. The univariate Cox regression analysis showed that PinX1 expression was an independent prognostic marker for ccRCC patients overall survival (hazard ratio, 0.628; 95% CI, 0.464–0.850; *P* = 0.003; Supplementary Table S1), and disease-specific survival (hazard ratio, 0.600; 95% CI, 0.433–0.832; *P* = 0.002; Supplementary Table S1). In multivariate Cox regression analysis, we found that PinX1 expression was also an independent prognostic marker for 5-year overall survival (hazard ratio, 0.640; 95% CI, 0.469–0.874; *P* = 0.005; Supplementary Table S2) and disease-specific survival (hazard ratio, 0.611; 95% CI, 0.436–0.857; *P* = 0.004; Supplementary Table S2). Because 5-year patients’ survival is widely used to predict outcome in ccRCC patients, our results clearly indicated that low PinX1 expression is associated with poor prognosis, suggesting that PinX1 may serves as a molecular prognostic marker for this aggressive disease.

### PinX1 suppresses migration and invasion of human ccRCC cells *in vitro*

**Figure 2:**
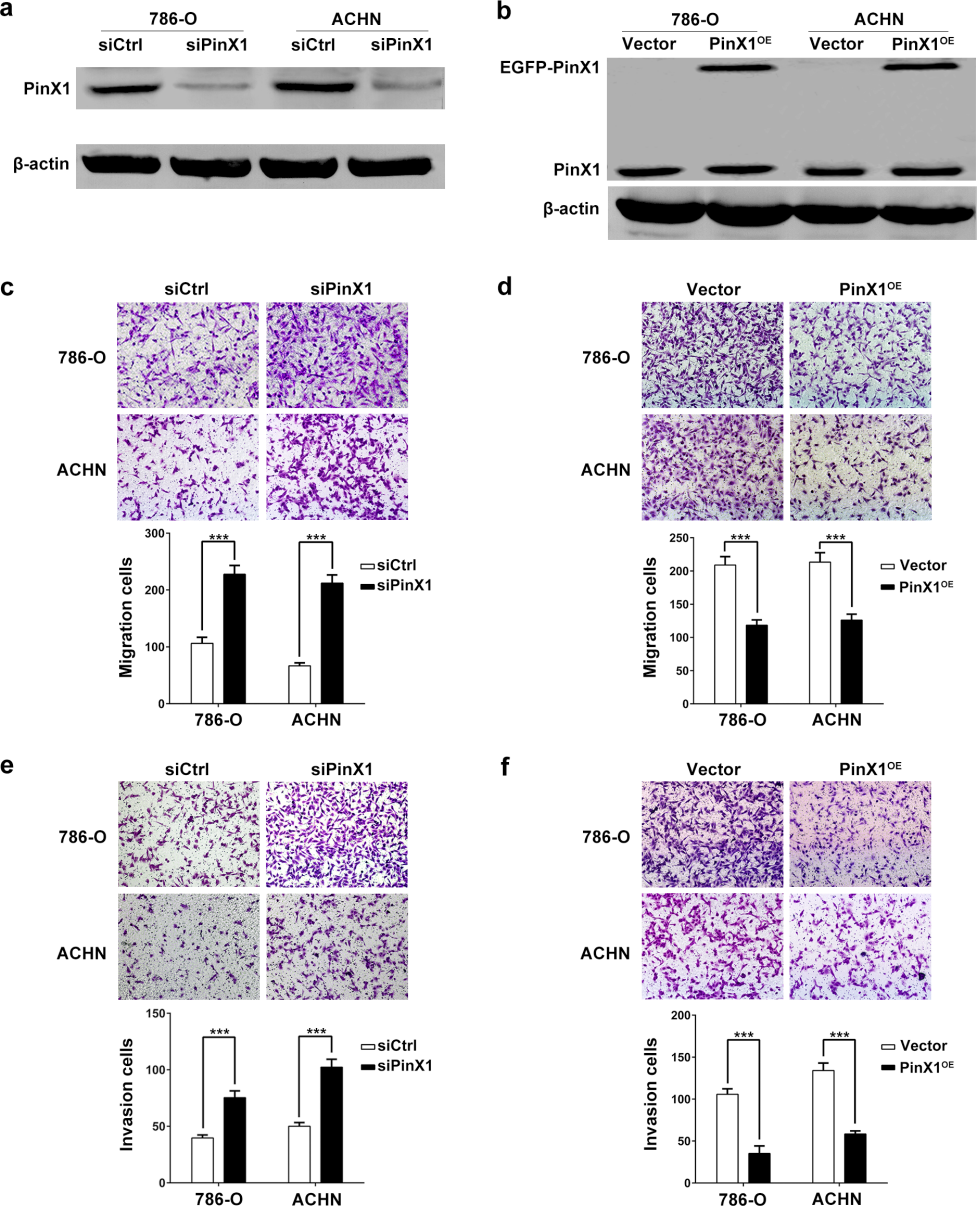
PinX1 inhibits migration and invasion of ccRCC cells **a.** Western blot analysis of the relative protein level of PinX1 in PinX1 knockdown (siPinX1) and control siRNA (siCtrl) groups for both 786-O and ACHN cell lines. It showed that PinX1 expression was significantly suppressed by PinX1 siRNA. **b.** Western blot analysis of the relative protein level of PinX1 in PinX1 overexpression (PinX1^OE^) and control vector (Vector) groups for both 786-O and ACHN cell lines. Ectopic PinX1 expression was detected apparently after cells transiently transfected with pEGFP-C3-PinX1 plasmid. **c.** and **e.** PinX1 knockdown significantly inhibited migration and invasion of 786-O and ACHN cells (2.5 × 10^4^ cells were seeded for migration assay and 2 × 10^4^ cells were seeded for invasion assay). **d.** and **f.** PinX1 overexpression significantly inhibited migration and invasion of 786-O and ACHN cells (3.5 × 10^4^ cells were seeded for migration assay and 3 × 10^4^ cells were seeded for invasion assay). All experiments were carried out in triplicate. Histograms represent means ± SD. ***, *P* < 0.001.

Because low PinX1 expression is associated with poor prognosis, supporting PinX1 may play important roles in one or more steps of tumor metastasis. Due to migration and invasion ability is crucial for tumor metastasis [28], we examined the effects of PinX1 on migration and invasion of ccRCC cells. We transiently transfected 786-O and ACHN cells with pEGFP-C3-control and pEGFP-C3-PinX1 plasmids or control siRNA and PinX1 siRNA, respectively. Twenty-four hours or forty-eight hours after transfection, PinX1 proteins were significantly overexpressed or knockdown in ccRCC cancer cells, respectively (Figure 2a and 2b). In the migration assay, we found that PinX1 knockdown in ccRCC cells significantly enhanced the ability to migrating through transwell filter inserts respectively (Figure 2c). Inversely, PinX1 overexpression dramatically suppressed the migration ability of ccRCC cells (Figure 2d). In cell invasion assay, we got the similar conclusion: PinX1 knockdown or overexpression can enhance or suppress the invasion ability of ccRCC cells (Figure 2e and 2f). However, overexpression or silence of PinX1 had no effect on the proliferation of ccRCC cells (Supplementary Figure S1a – S1d).

### PinX1 inhibits human ccRCC cells’ migration and invasion abilities by suppressing MMP-2 expression and activity

**Figure 3:**
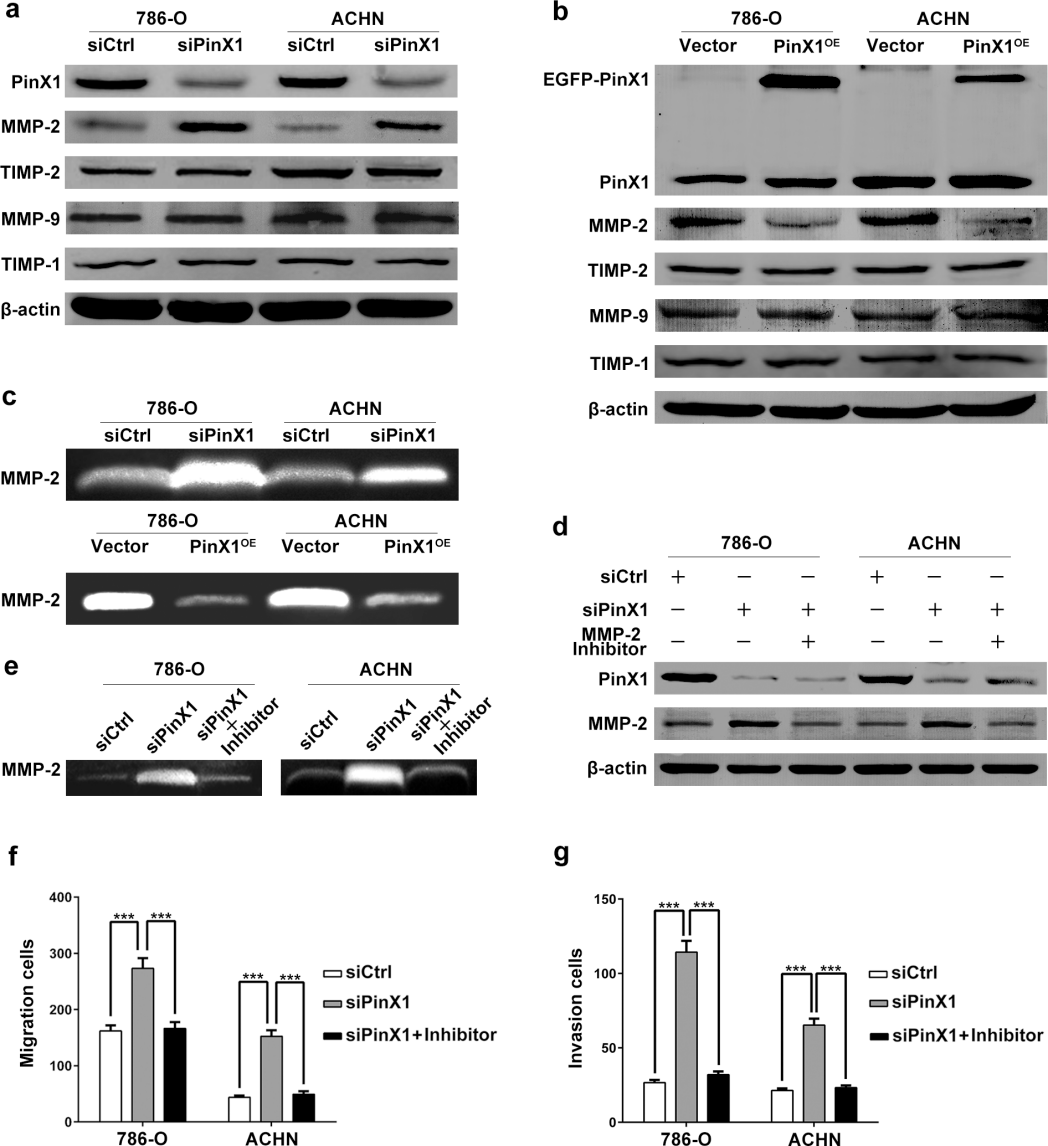
PinX1 inhibits migration and invasion of ccRCC cells by suppressing MMP-2 expression and activity **a.** Western blotting of PinX1, MMP-2, MMP-9, TIMP-1 and TIMP-2 from ccRCC cells transfected with the PinX1 siRNA or control siRNA. MMP-2 expression was up-regulated independent of TIMP-2 in PinX1 knockdown ccRCC cells. **b.** Western blotting of PinX1, MMP-2, MMP-9, TIMP-1 and TIMP-2 from ccRCC cells transfected with the pEGFP-C3-PinX1 plasmid or vector control. MMP-2 expression was down-regulated independent of TIMP-2 in PinX1 overexpression ccRCC cells. **c.** top panel, Gelatin zymography analysis of the enzyme activity of MMP-2 in PinX1 knockdown and control group for both 786-O and ACHN cell lines (gels were incubated for 10 h for 786-O cells and 48 h for ACHN cells). Bottom panel, Gelatin zymography analysis of the enzyme activity of MMP-2 in PinX1 overexpression and control group for both 786-O and ACHN cell lines (gels were incubated for 16 h for 786-O cells and 96 h for ACHN cells). The MMP-2 enzyme activity was significantly enhanced after PinX1 overexpressing in ccRCC cells, conspicuously suppressed after PinX1 knockdown. **d.** Western blotting of PinX1 and MMP-2 from ccRCC cells transfected with the control siRNA, PinX1 siRNA or co-treated with MMP-2 selective inhibitor I (10 μM). The enhancement of MMP-2 expression regulated by PinX1 knockdown in ccRCC cells was blocked by MMP-2 inhibitor I. **e.** Gelatin zymography analysis of the enzyme activity of MMP-2 in ccRCC cells transfected with the PinX1 siRNA or co-treated with MMP-2 selective inhibitor I (10 μM). The enhancement of MMP-2 activity regulated by PinX1 knockdown in ccRCC cells was blocked by MMP-2 inhibitor. **f.** and **g.** The enhancement of migration and invasion regulated by PinX1 knockdown in ccRCC cells was blocked by MMP-2 inhibitor I. All experiments were carried out in triplicate. Histograms represent means ± SD. ***, *P* < 0.001.

The matrix metalloproteinase (MMP) family can degrade the extracellular matrix (ECM) in the major early stages of a number of malignant tumors, which plays an important role in cancer invasion and metastasis [29]. To investigate the mechanisms of PinX1 regulating migration and invasion in ccRCC cells, we performed western blot and gelatin zymography to detect the MMPs protein levels and activities in 786-O and ACHN cells. Our data showed that the MMP-2 expression and activity were negatively regulated by PinX1 in ccRCC cells, but not MMP-9 (Figure 3a–3c). So we supposed PinX1 suppress migration and invasion of ccRCC cells by regulating MMP-2 expression and activity. To further validate our hypothesis, we added MMP-2 selective inhibitor I(sc-204092, Santa Cruz) at the same time of PinX1 siRNA transfecting into ccRCC cells. As expected, the up-regulation of MMP-2 expression and activity was blocked by MMP-2 selective inhibitor I (Figure 3d and 3e). We also validated this phenomenon by migration and invasion analysis. The migration and invasion ability can be enhanced by inhibiting of PinX1 in ccRCC cells, however, these regulations were blocked by MMP-2 selective inhibitor I. Above all, it was confirmed that PinX1 inhibited ccRCC cells’ migration and invasion by suppressing MMP-2 expression and activity (Figure 3f and 3g).

We know that tissue inhibitors of matrix metalloproteinases (TIMPs) have the ability to inhibit the catalytic activity of MMPs, and the imbalance between MMPs and TIMPs is responsible for cancer metastasis [30]. TIMP-1 and TIMP-2 is the tissue inhibitor of MMP-9 and MMP-2. In order to understand whether PinX1 regulates MMP-2 expression and activity by TIMP-2, we detected the expression of TIMP proteins. Unfortunately, data showed that TIMP-2 expression never changed when MMP-2 expression was up-regulated or down-regulated corresponded with PinX1 knockdown or overexpression. TIMP-1 expression was also not changed as well as MMP-9 expression. (Figure 3a and 3b). These clinical data urged us to investigate the potential mechanism of PinX1 regulating MMP-2 expression and activity.

### PinX1 suppresses MMP-2 expression via NF-κB-dependent transcription

**Figure 4:**
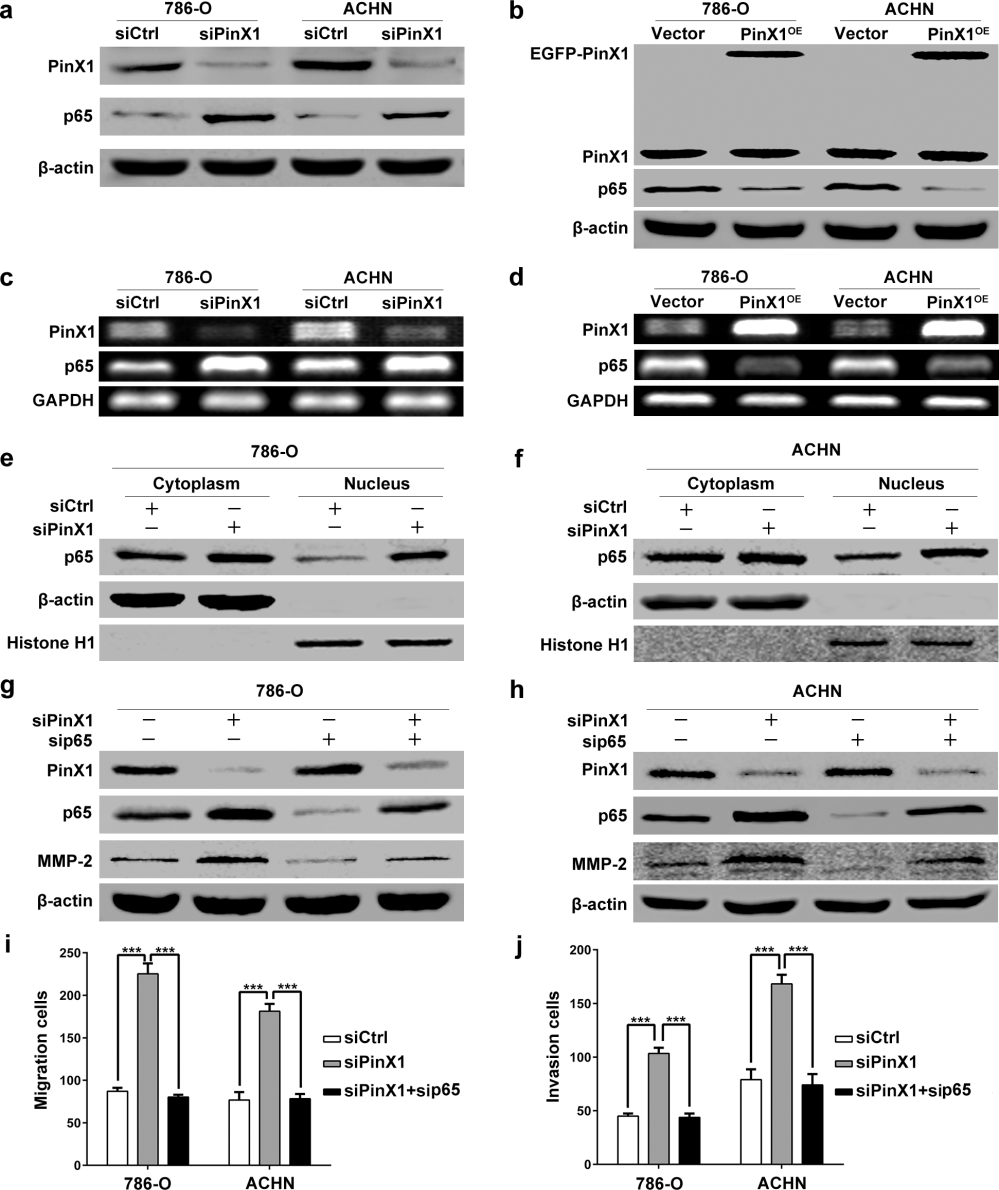
PinX1 inhibits migration and invasion of ccRCC cells by suppressing MMP-2 expression via NF-κB-dependent transcription **a.** Western blotting of PinX1 and p65 from ccRCC cells transfected with the PinX1 siRNA or control siRNA. p65 expression was up-regulated in PinX1 knockdown ccRCC cells. **b.** Western blotting of PinX1 and p65 from ccRCC cells transfected with the pEGFP-C3-PinX1 plasmid or vector control. p65 expression was down-regulated in PinX1 overexpression ccRCC cells. **c.** RT-PCR determined mRNA levels of PinX1 and p65 in ccRCC cells transfected with PinX1 siRNA or control siRNA. The mRNA level of p65 was increased in PinX1 knockdown ccRCC cells. **d.** RT-PCR determined mRNA levels of PinX1 and p65 in ccRCC cells transfected with pEGFP-C3-PinX1 plasmid or vector control. The mRNA level of p65 was decreased in PinX1 overexpression ccRCC cells. **e** and **f.** Western blotting determined cellular distribution of p65 in 786-O and ACHN cells transfected with control siRNA and PinX1 siRNA. Inhibition of PinX1 significantly increased the nuclear accumulation of p65. **g.** and **h.** Western blotting of PinX1, p65 and MMP-2 from ccRCC cells transfected with the control siRNA, PinX1 siRNA, p65 siRNA or co-treated with PinX1 siRNA and p65 siRNA. MMP-2 expression was inhibited by p65 siRNA as well as p65 expression. The enhancement of MMP-2 expression regulated by PinX1 knockdown as well as p65 in ccRCC cells was abolished by p65 siRNA. **i.** and **j.** The enhancement of migration and invasion regulated by PinX1 knockdown in ccRCC cells was abolished by p65 siRNA. All experiments were carried out in triplicate. Histograms represent means ± SD. ***, *P* < 0.001.

NF-κB is a critical transcription factor activated in a large number of human cancers and plays a crucial role in tumor development and progression [31]. It regulates downstream genes associated with proliferation, survival, angiogenesis and metastasis [31, 32]. Its κB site was identified in the promoters of genes that encode MMP-2 [32], and the activation of NF-κB induces membrane type proteases (MT1-MMP), the activator of pro-MMP-2 which proteolytically cleaves to generate functionally active MMP-2 [33]. Therefore, we presumed that PinX1 regulated MMP-2 expression via NF-κB signaling pathway. To test this hypothesis, we first determined the protein and mRNA levels of p65 in 786-O and ACHN cells after PinX1 overexpression or knockdown. Western blot results showed that the level of p65 protein was increased sharply in ccRCC cells after PinX1 knockdown (Figure 4a). In contrast, p65 expression dramatically down-regulated in ccRCC cells after PinX1 overexpression (Figure 4b). RT-PCR showed that the level of p65 mRNA was increased or decreased significantly in ccRCC cells after PinX1 knockdown or overexpression (Figure 4c and 4d). Moreover, we determined the cellular distribution of p65 in ccRCC cells by immunoblot after PinX1 knockdown. Inhibition of PinX1 significantly increased the nuclear distribution of p65 (Figure 4e and 4f). Together, these data indicate that inhibition of PinX1 promotes p65 expression and nuclear localization, activates NF-κB-induced transcription.

To further confirm whether NF-κB activation induced by PinX1 inhibition caused the up-regulation of MMP-2 in human ccRCC cells, Western blot analysis showed that p65 siRNA can inhibited the expression of p65 and MMP-2 in ccRCC cells (Figure 4g and 4h). The level of MMP-2 protein was up-regulated by PinX1 suppression in ccRCC cells, but these effects were further blocked by knockdown of p65 expression with a specific siRNA (Figure 4g and 4h). These data provided further evidence that PinX1 might regulate MMP-2 expression via NF-κB transcription factor.

We also validated the mechanism by migration and invasion analysis. The migration and invasion ability can be enhanced by PinX1 inhibition in ccRCC cells, however, these effects were abolished by co-suppressing PinX1 and p65 in ccRCC cells (Figure 4i and 4j).

### PinX1 suppresses ccRCC cells metastasis *in vivo*

**Figure 5:**
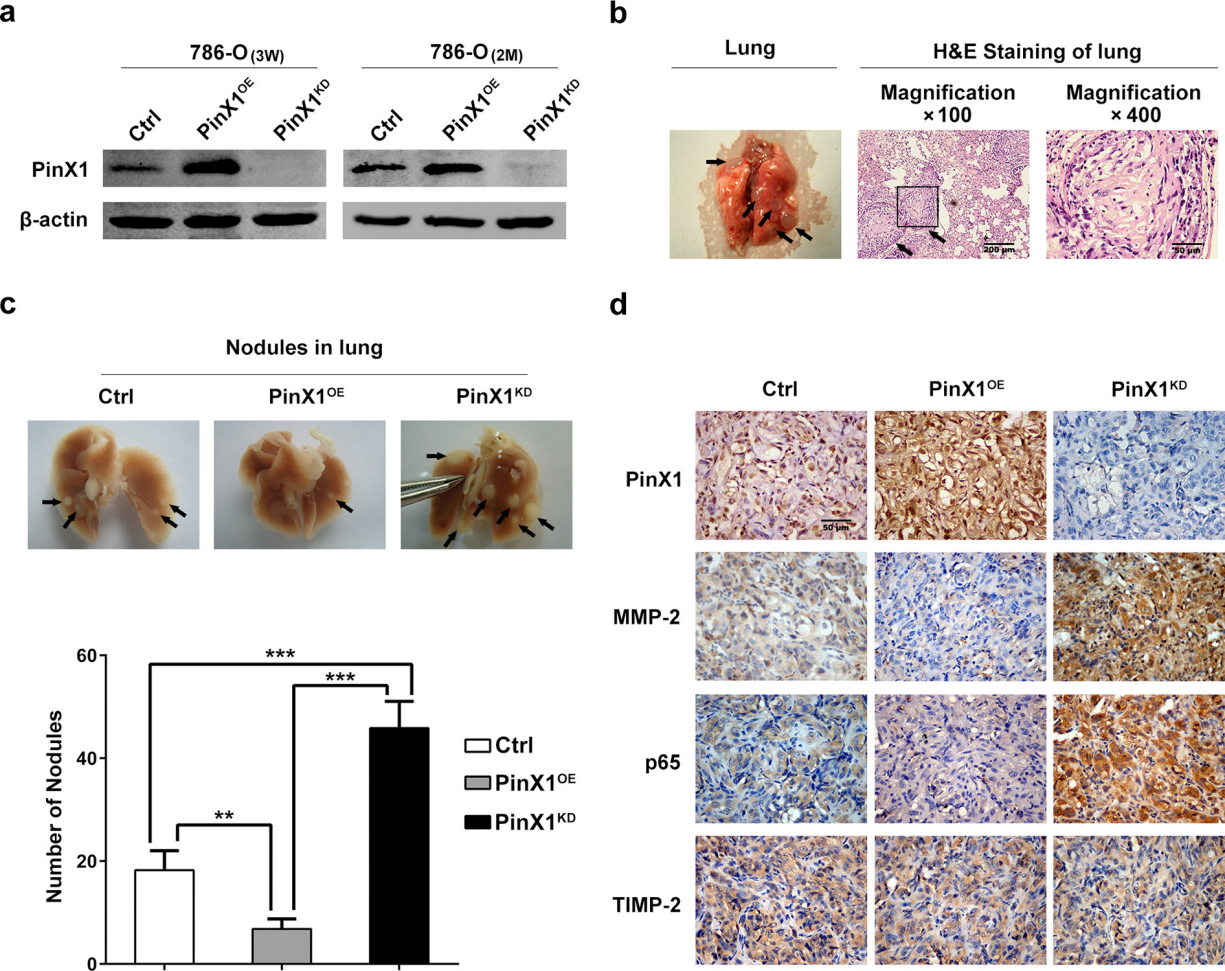
PinX1 suppress ccRCC metastasis *in vivo* **a.** left panel, Western blotting of PinX1 from PinX1^OE^-786-O cell lines, PinX1^KD^-786-O cell lines and Ctrl-786-O cell lines selected with puromycin for 3 weeks after lentivirus infection. Right panel, PinX1 expression levels were not changed in PinX1^OE^, PinX1^KD^ and Ctrl 786-O cell lines without puromycin selection for 2 months. **b.** Representative image of lung with metastatic nodules (left panel) and H&E staining sections of lung (right panel) 2 months after injection of PinX1^KD^ 786-O cell lines in BALB/c nude mouse through tail vein. Arrows indicate metastatic nodules. **c.** top panel, Representative images of 10% buffered formalin fixed lungs with metastatic nodules 2 months after respective injection of Ctrl, PinX1^OE^ and PinX1^KD^ 786-O cell lines. Arrows indicate metastatic nodules. Bottom panel, the number of lung metastatic nodules was counted under a dissecting microscope. A statistically dramatic increase in the number of the lung metastases was seen in PinX1^KD^ group, compared with the PinX1^OE^ group and these two groups also had significant diversity compared with Ctrl group respectively. Data are displayed with means ± SD from 12 mice in each group. **, *P* < 0.01; ***, *P* < 0.001. **d.** Immunostaining of PinX1, MMP-2, p65 and TIMP-2 in metastatic nodules of PinX1^OE^, PinX1^KD^ and Ctrl 786-O groups. MMP-2 and p65 expression in PinX1^OE^ group were much lower compared with PinX1^KD^ group and Ctrl group but TIMP-2 expression was not change in every group.

To further address the functional role of PinX1 in ccRCC metastasis *in vivo*, PinX1^OE^-786-O cell lines, PinX1^KD^-786-O cell lines and Ctrl-786-O cell lines were established. After 3 weeks selection following with lentivirus infection, the PinX1 protein levels of these cell lines were confirmed by western blot (Figure 5a, left panel). To detect whether the levels of PinX1 protein expression in these three cell lines could be changed without puromycin selection lasting 2 months, we incubated them without adding puromycin for 2 months *in vitro*. Then the expression of PinX1 of these three cell lines were detected by western blot, it was shown that the levels of PinX1 proteins expression had not been changed without puromycin selection for 2 months (Figure 5a, right panel).

Three groups of nude mice were injected through tail vein with PinX1^OE^-786-O, PinX1^KD^-786-O and Ctrl-786-O cells respectively. After 2 months, three groups of mice were sacrificed and their lungs were resected (Figure 5b, left panel) and fixed in 10% buffered formalin for metastatic nodules counting and further histopathological analysis. Randomly selected metastatic nodules had been validated by H&E staining (Figure 5b, right panel). Extensive tumor formation was found in PinX1^KD^ group. In contrast, the lungs in PinX1^OE^ group had fewer and smaller detectable tumor nodules (Figure 5c, top panel). A statistically dramatic increase in the number of the lung metastases was seen in PinX1^KD^ group, compared with the PinX1^OE^ group and these two groups also had significant diversity compared with Ctrl group respectively (Figure 5c, bottom panel).

Immunohistochemical staining of metastatic nodules in lungs resected from nude mice showed that MMP-2 and p65 expression in PinX1^OE^ group were much lower compared with PinX1^KD^ group and Ctrl group but TIMP-2 expression was not change in every group (Figure 5d). These had further validated our conclusion which had been demonstrated previously *in vitro*.

## DISCUSSION

PinX1 is a novel cloned gene localized at human chromosome 8p23 which is frequently associated with loss of heterozygosity in a variety of human malignancies. Increasing evidence demonstrate that reduced expression of PinX1 promotes cancer progression and propose that PinX1 may be an attractive therapeutic target for human cancers [34]. However, the PinX1 expression status and its correlation with the clinicopathological features in ccRCC have never been investigated. In the present study, two independent ccRCC cohorts TMA were studied. Our data showed that PinX1 expression was apparently decreased in ccRCC tissues compared with normal renal tissues and paired adjacent non-tumor tissues (Figure 1a and 1b). We also demonstrated that lower PinX1 staining was significantly correlated with advanced stages and worse survival in ccRCC patients (Figure 1c and 1d; Table 1). Moreover, univariate and multivariate Cox proportional hazards regression analysis investigated that low PinX1 expression was a strong independent negative prognostic indicator for clear cell renal cell carcinoma (Supplementary Table S1, Table S2). These findings indicate that PinX1 may be involved in the progression of ccRCC and be a significant prognostic factor for ccRCC patients.

Management of metastatic clear cell renal cell carcinoma in clinical is always a difficult problem to solve. The natural history of ccRCC may be unpredictable. For example, between 4.2% and 7.1% of patients with tumors ≤ 4 cm that are usually indolent harbor metastatic disease at presentation and are at an elevated risk of disease-specific mortality [35]. Conversely, as many as 40% of patients with lymph node metastases diagnosed at nephrectomy are alive 5 years after surgery [36]. Thus, a number of independent prognostic markers have been searched and validated such as Ki-67, p53, serum CAIX [37]. In this study, we revealed a new potential independent negative prognostic factor for ccRCC. Although it needs more clinic trials to validate this finding, we hope it is beneficial to improve the prognostic accuracy for ccRCC patients.

Our clinical data urged us to carry out a series of *in vitro* and *in vivo* experiments to explore the potential mechanisms. The metastasis process is thought to involve a series of interdependent events. These include the attachment of the tumor cells to the receptors within the basement membrane [38], degradation of extracellular matrix (ECM) via matrix metalloproteinases (MMPs) [39], and, finally, the migration of cancer cells into the target organ tissue react to specific chemotactic stimuli [40] to form secondary tumors. Therefore, the invasion and migration are crucial for tumor metastasis and MMPs is essential for invasion process. Our data demonstrated that PinX1 inhibited ccRCC cells’ migration and invasion abilities by down-regulating MMP-2 expression and activity *in vitro* (Figures 2, 3). The catalytic activity of MMP-2 is controlled by interaction with the TIMP-2. But in our study, TIMP-2 expression was not seemed to be the regulator of MMP-2 activation in ccRCC cells (Figure 3a and 3b). Therefore, we turned to NF-κB, another common regulator of MMP-2.

Accumulating evidence demonstrate that several MMPs (including MMP-2) expression and activation were regulated by p65 subunit up-regulation and nuclear translocation induced NF-κB activation in many human cancers [41–45]. The present results showed that down-regulation of PinX1 by siRNA significantly enhanced the protein and mRNA expression of p65, while overexpression of PinX1 induced down-regulation of p65 mRNA and protein levels (Figure 4a–4d). Inhibition of PinX1 also significantly increased p65 nuclear accumulation (Figure 4e and 4f), activated NF-κB-induced transcription. Moreover, the p65 specific siRNA markedly prevented MMP-2 expression induced by knockdown of PinX1 (Figure 4g and 4h). The positive regulation of migration and invasion induced by PinX1 knockdown in ccRCC cells was also be suppressed by inhibition of p65 expression (Figure 4i and 4j). Thus, these findings suggested that PinX1 regulated ccRCC cells migration and invasion through a process involving NF-κB-dependent regulation of MMP-2. It is the first time to validate PinX1 involves in regulating cancer metastasis independent of its ordinary roles like maintaining telomerase activity, the length of telomerase and chromosome stability.

**Figure 6:**
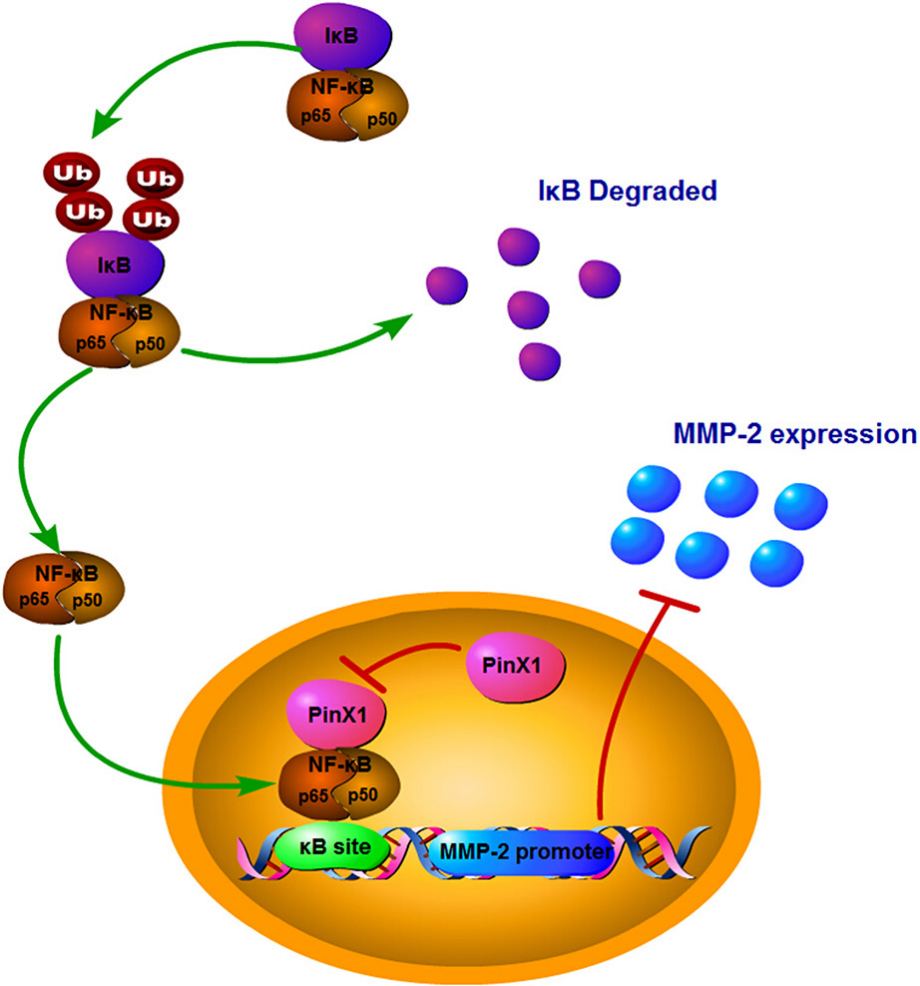
A hypothetic model of PinX1 suppresses MMP-2 expression via NF-κB signaling pathway We presume that PinX1 can inhibit the expression of MMP-2 owing to the suppression of transcriptional activity of NF-κB proteins by direct protein-protein interaction with its G-patch domain.

We regret to investigate the potential regulation mechanism between PinX1 and NF-κB pathway, the regulation of NF-κB by PinX1 direct or indirect is not clear. however, we have found some conceivable relations between them. Firstly, in 2004, Wang et al had reported that there were several putative binding sites for transcription factors such as CREB, p53, E2F, GATA-1, USF, HNF, NF-κB and C/EBP at the promoter region of human PinX1 gene [46]. Recently, it had been demonstrated that PinX1 expression was directly activated by P53 in cervical cancer cells [47]. So we presume that NF-κB also can directly activates PinX1 expression, and p65 was regulated by PinX1 via negative feedback. On the other hand, Human PinX1 contains a Gly-rich patch (G-patch) domain at its N-terminal and TID domain (telomerase inhibitory domain) at its C-terminal [15]. G-patch domain was firstly reported by L. Aravind et al in 1999 [48], but the function of its G-patch domain has been researched rarely. It exists in a number of putative RNA-binding proteins involved in tumor suppression and DNA-damage repair. Moreover, it is an important nucleic acids binding domain at the C-terminus of the NF-κB-repression factor (NRF) [49]. NRF can inhibit the transcriptional activity of NF-κB proteins by direct protein-protein interaction [50, 51]. Thus, we presume that PinX1 also can inhibit the transcriptional activity of NF-κB proteins by direct protein-protein interaction with its G-patch domain (Figure 6). These hypothesizes have never been validated. However, the connection between in ccRCC cells exists indeed. So we will investigate the potential molecular mechanisms between PinX1 and NF-κB signaling pathway in ccRCC cells continually.

*In vivo* study investigated that PinX1 overexpression in ccRCC cells significantly inhibited the formation of metastasis nodules in lung of nude mice while PinX1 knockdown dramatically enhanced the metastasis process (Figure 5c). Moreover, trends of p65 and MMP-2 immunostaining in metastasis nodules of PinX1^KD^ and PinX1^OE^ groups were coincident with *in vitro* experiments. It confirmed that PinX1 suppressed ccRCC invasion and metastasis by inhibiting MMP-2 expression and activity via NF-κB pathway.

Taken together, based on these findings and combined with the fact that metastasis is the major cause of ccRCC patient death, we can conclude that loss of PinX1 expression was significantly correlated with ccRCC progression and was an independent prognostic factor of worse outcome in ccRCC patients and restoration of PinX1 may be a novel strategy for aggressive human ccRCC. Although tyrosine kinase inhibitors (TKI) became the most successful class of drugs in the treatment of metastatic ccRCC, there was approximately 25% of the patients had intrinsic resistance to first line TKIs therapy [52]. Our results firstly provided the *in vitro* and *in vivo* evidences that targeting PinX1 might represent a new therapy to suppress ccRCC metastasis. We hope these findings might have shed light on future directions for identification of novel biomarkers for ccRCC and the development of targeted drugs.

## MATERIALS AND METHODS

### Patients and specimens

Two independent ccRCC cohorts tissue microarray (TMA) were utilized in this study. The training cohort TMA was purchased from Shanghai Xinchao Biotechnology (Shanghai, China). It included 75 patients who underwent Radical nephrectomy from 2006 to 2008. The ccRCC tissues and paired non-cancerous tissues from these patients were obtained. The array dot diameter was 1.5 mm, and each dot represented a tissue spot from one individual specimen that was selected and pathologically confirmed.

The validation cohort TMA consisted of 278 surgical cases and 35 cases of normal renal tissues was constructed by a contract service at the National Engineering Centre for Biochip (Shanghai, China). Patients with ccRCC who underwent Radical nephrectomy without prior treatment were recruited from Affiliated Hospital of Xuzhou Medical College, between 2005 and 2008. The patients’ clinicopathologic information including age at diagnosis, sex, tumor diameter, depth of invasion, lymph node metastasis, and TNM stage was obtained from the Medical Record of the Affiliated Hospital of Xuzhou Medical College. 5-year Clinical follow-up results were available for 243 patients from the Xuzhou area. All the tissue specimens were obtained for the present research with patients’ informed consent, and the use of human specimens was approved by the Review Board of the Affiliated Hospital of Xuzhou Medical College.

### Immunohistochemistry

Immunohistochemistry was performed as described before [53]. According to the streptavidin-peroxidase (Sp) method using a standard Sp Kit (Zhongshan biotech, Beijing, China). TMA slides were dewaxed at 60°C for 20 minutes followed by two 10-minute washes with xylene and then rehydrated with graded ethanol and distilled water. Endogenous peroxidases were inhibited by 3% H_2_O_2_ for 30 minutes. Antigen retrieval was performed in a microwave oven with 10 mM citrate buffer (pH 6.0) at 95°C for 30 minutes. After 30-minute blocking with 5% normal goat serum, the sections were incubated with polyclonal rabbit anti-PinX1 antibody (1:50 dilution; Novus Biologicals, Littleton, CO, USA) overnight at 4°C. The slides were then incubated for 1 hour with a biotin-labeled secondary antibody, followed by avidin-peroxidase reagent and 3, 3′-diaminobenzidine (DAB; Zhongshan biotech, Beijing, China) substrate. After hematoxylin counterstain and Dehydration, the sections were sealed with cover slips. Negative controls were performed by Phosphate buffered saline (PBS) replaced PinX1 antibody during the primary antibody incubation. The staining of the normal renal tissues in each microarray slide was evaluated as the quality control of the immunostaining.

### Evaluation of immunostaining

Positive PinX1 immunostaining is defined mainly in the nucleus area and also can be observed in the cytoplasm. We grade it according to both the intensity and percentage of cells with positive staining. The immunoreactivity was assessed by 2 pathologists simultaneously, and a consensus was reached for each core. The staining intensity of PinX1, p65, et al proteins involved in our study was scored 0 to 3 (0 = negative; 1 = weak; 2 = moderate; 3 = strong). The percentage of protein-positive stained cells was also scored into 4 categories: 1 (0–25%), 2 (26%–50%), 3 (51%–75%), and 4 (76%–100%). In the cases with a discrepancy between duplicated cores, the average score from the 2 tissue cores was taken as the final score. The level of relevant proteins staining was evaluated by immunoreactive score (IRS), which is calculated by multiplying the scores of staining intensity and the percentage of positive cells. Based on the IRS, staining pattern was defined as negative (IRS: 0), weak (IRS: 1–3), moderate (IRS: 4–6), and strong (IRS: 8–12).

### Animals and cell lines

Female BALB/c nude mice, 6 weeks old, were purchased from the Shanghai Laboratory Animal Center (Shanghai, China) for studies approved by the Animal Care Committee of Xuzhou Medical College. Human clear cell adenocarcinoma cell line 786-O and ACHN were purchased from the Shanghai Institute of Biochemistry and Cell Biology, Chinese Academy of Sciences (Shanghai, China). Cells were cultured as described before [54]. 786-O cells were cultured in Roswell Park Memorial Institute 1640 medium (RPMI1640; Invitrogen, Shanghai, China) supplemented with 10% fetal calf serum (Invitrogen), ACHN cells were cultured in Minimum Essential Media medium (MEM; Invitrogen) supplemented with 10% fetal calf serum. These two cells were both incubated in a 37ºC humidified incubator with 5% CO2.

### DNA and siRNA transfections, and stable cell line generation

The pEGFP-C3 and pEGFP-C3-PinX1 expression plasmids were obtained from Dr Xiao-Fen Lai (Southern Medical University, Guangzhou, China). The PinX1 siRNA and scrambled siRNA were purchased from GenePharma (Shanghai, China). NF-κB-p65 siRNA was purchased from Santa Cruz Biotechnology (Santa Cruz, CA, USA). Transfection of the pEGFP-C3-PinX1 plasmid and the pEGFP-C3 vector into the 786-O and ACHN cells were carried out using Lipofectamine 2000 transfection reagent (Invitrogen) following the manufacturer's protocol. PinX1 siRNA, NF-κB-p65 siRNA or scrambled siRNA was transfected into the 786-O and ACHN cells by siLentFect Lipid Reagent (Bio-Rad, Hercules, CA, USA) according to the manufacturer's instructions. The PinX1 overexpression 786-O cell lines (PinX1^OE^-786-O), PinX1 knockdown 786-O cell lines (PinX1^KD^-786-O) and control 786-O cell lines (Ctrl-786-O) were established by infecting with lentivirus packing PinX1 expression vector (EGFP is not fused with PinX1 in this vector), PinX1 shRNA expression vector and control vector respectively (GenePharma, Shanghai, China). Target cells were infected with lentivirus for 48 hours then selected with puromycin (Santa Cruz) for 3 weeks.

### Cell migration and invasion assay

Cell migration and invasion assay were performed using modified two chamber plates with a pore size of 8 μm. The transwell filter inserts with or without Matrigel (BD Biosciences) coating were used respectively for invasion and migration assay. The detailed conditions are described previously [54].

### Gelatin zymography

Gelatin zymography was performed as described before [55]. It was used to detect MMP-2 and MMP-9 activity. 2.5 × 10^6^ cells were seeded in 100 mm plate for 24 h. The proteins in the conditioned medium were concentrated with Amicon Ultra-4–30 k centrifugal filters (Millipore, Billerica, MA, USA) at 7500 g for 20 min at 4°C. Twenty microgram of the proteins was loaded in nondenaturating conditions on a 10% polyacrylamide gel containing 0.1% gelatin (Sigma, St Louis, MO, USA). After electrophoresis, gels were swang in 2.5% Triton X-100 for 30 minutes with single change of detergent solution. Gels were incubated for 10 h or 16 h (for 786-O cells in RNAi group or overexpression group) and 48 h or 96 h (for ACHN cells in RNAi group or overexpression group) at 37°C in incubation buffer (50 mM Tris-HCl (pH 8.8), 5 mM CaCl_2_, 1 μM ZnCl_2_ and 0.02% NaN_3_), stained with 0.1% Coomassie brilliant blue R-250 (Sigma) at least for 2 h, and destained in 10% acetic acid and 45% methanol. Gelatinolytic activity was shown as clear areas in the gel. Gels were photographed and then quantitatively measured by scanning densitometry.

### RNA extraction, semiquantitative reverse transcription–PCR (RT-PCR)

Total RNA from cell lines was extracted using Trizol Reagent (Beyotime Biotechnology, Shanghai, China) according to the manufacturer's instructions. In semiquantitative reverse transcription–PCR experiments, total RNA (2 μg) was reverse transcribed using a BeyoRT cDNA First Strand cDNA synthesis kit (Beyotime Biotechnology, Shanghai, China) in a 20 μl reaction mixture containing 5× reverse transcriptase reaction buffer, 0.5 μg of oligo(dT)_18_ primer and 1 μl of BeyoRT M-MuLV reverse transcriptase, incubated for 60 min at 42°C and then heated for 10 min at 70°C. The mixture was heat inactivated at 94°C for 5 min, and 1 μl of the inactivated reverse transcriptase reaction mixture and 19 μl of the PCR mixture were combined and amplified according to a standard PCR reaction that is listed below. The sequences of the upstream and downstream primers used are as follows: PinX1: 5′- CAGTCACCCAGGTCCAGA- 3′ and 5′- CTTAGGCTGGAGGTAACTT - 3′; p65: 5′ - ACAACAACCCCTTCCAAGAAGA - 3′ and 5′- CAGCCTGGTCCCGTGAAATA - 3′; GAPDH: 5′ - CCCGGGATGCTAGTGCG - 3′ and 5′ – GCCCAATAC GACCAAATCAGA - 3′. PCR analysis was performed using the following conditions: the average temperature and cycles were 58°C for 35 cycles with the PinX1 primers, 56°C for 30 cycles with the p65 primers and 55°C for 30 cycles with the GAPDH primers. The amplified products were analyzed by agarose gel electrophoresis using a 2.5% gel and ethidium bromide staining.

### Nuclear cytoplasmic fractionation

Subcellular fractionation was performed with a Nuclear and Cytoplasmic Protein Extraction Kit (Beyotime Biotechnology, Shanghai, China). The detailed conditions are described previously [43]. Briefly, the cells were collected in ice-cold PBS, suspended in the hypotonic buffer, and incubated for 15 min on ice. The detergent was added, and the cells were vortexed for 5 s, and incubated for 1 min on ice, followed by centrifugation at 16,000 rpm for 5 min at 4°C. The cytoplasmic fraction was collected into separate tubes. The nuclear fraction was lysed in the complete lysis buffer on ice for 30 min, centrifuged at 16,000 rpm for 10 min at 4°C, and transferred into separate tubes.

### Antibodies and western blot (WB)

Antibodies against the following proteins were used: PinX1 (1:200 for WB, 1:50 for IHC; Novus Biologicals); MMP-2 (1:100 for WB; Cell Signaling Technology, Beverly, MA, USA); MMP-2 (1:50 for IHC; Santa Cruz); MMP-9 (1:200 for WB, Cell Signaling Technology); TIMP-1(1:200 for WB, Santa Cruz); TIMP-2 (1:200 for WB, 1:100 for IHC; Santa Cruz); NF-κB-p65 (1:500 for WB, 1:200 for IHC; Santa Cruz); β-actin (1:1000 for WB; Cell Signaling Technology); Infrared IRDye-labeled secondary antibody (1:10000; LI-COR, Lincoln, NE, USA) was applied to the blot for 1 hour at room temperature. The signals were detected with Odyssey Infrared Imaging system (LI-COR).

Western blot analysis was performed as described previously [56]. Cells were harvested and washed twice with PBS. Whole-cell proteins were extracted as described previously. Protein concentrations were determined by protein assay (Bio-Rad). All protein samples were denatured, electrophoresed on SDS/polyacrylamide gels and transferred onto polyvinylidene difluoride membranes (Millipore).

### Tail vein metastasis assay

To produce experimental metastasis, the BALB/c nude mice were randomly divided into three groups consisting of 12 mice each. PinX1^OE^-786-O, PinX1^KD^-786-O and Ctrl-786-O cells were suspended in PBS. The mice were injected intravenously with 2.5 × 10^6^ 786-O cells in 0.2 ml of PBS through tail vein. After 2 months, the three groups of mice were sacrificed, their lungs were resected and fixed in 10% buffered formalin for metastatic nodules counting and further histopathological analysis. The number of metastatic nodules presented on the surface of each set of lungs was counted by visual inspection using a stereoscopic dissecting microscope.

### Statistical analysis

Data are expressed as the means ± SD. Two-factor analysis of variance procedures and the Dunnett's *t*-test were used to assess differences within treatment groups. For TMA, statistical analysis was performed with SPSS 20 software (SPSS, Inc, Chicago, IL). The association between PinX1 staining and the clinicopathologic parameters of the ccRCC patients, including age, gender, tumor size, pT status, pN status and TNM stage, was evaluated by two sided Fisher's exact tests. Differences in IRS for PinX1 staining in primary tumors and their paired adjacent normal renal tissues were assessed by the Wilcoxon test (grouped). The Kaplan-Meier method and log-rank test were used to evaluate the correlation between PinX1 expression and patient survival. Cox regression model was used for multivariate analysis. Differences were considered significant when *P* < 0.05.

## SUPPLEMENTARY FIGURE AND TABLES



## References

[1] Siegel R, Desantis C, Jemal A (2014). Colorectal cancer statistics, 2014. CA: a cancer journal for clinicians.

[2] Baldewijns MM, van Vlodrop IJ, Schouten LJ, Soetekouw PM, de Bruine AP, van Engeland M (2008). Genetics and epigenetics of renal cell cancer. Biochimica et biophysica acta.

[3] Brugarolas J (2007). Renal-cell carcinoma—molecular pathways and therapies. The New England journal of medicine.

[4] Cohen HT, McGovern FJ (2005). Renal-cell carcinoma. The New England journal of medicine.

[5] Shay JW, Wright WE (2002). Telomerase: a target for cancer therapeutics. Cancer cell.

[6] Blackburn EH (2001). Switching and signaling at the telomere. Cell.

[7] Kim NW, Piatyszek MA, Prowse KR, Harley CB, West MD, Ho PL, Coviello GM, Wright WE, Weinrich SL, Shay JW (1994). Specific association of human telomerase activity with immortal cells and cancer. Science.

[8] Broccoli D, Young JW, de Lange T (1995). Telomerase activity in normal and malignant hematopoietic cells. Proceedings of the National Academy of Sciences of the United States of America.

[9] Wang J, Xie LY, Allan S, Beach D, Hannon GJ (1998). Myc activates telomerase. Genes & development.

[10] Lin SY, Elledge SJ (2003). Multiple tumor suppressor pathways negatively regulate telomerase. Cell.

[11] Smogorzewska A, de Lange T (2004). Regulation of telomerase by telomeric proteins. Annual review of biochemistry.

[12] van Steensel B, de Lange T (1997). Control of telomere length by the human telomeric protein TRF1. Nature.

[13] de Lange T (2005). Shelterin: the protein complex that shapes and safeguards human telomeres. Genes & development.

[14] Lee TH, Tun-Kyi A, Shi R, Lim J, Soohoo C, Finn G, Balastik M, Pastorino L, Wulf G, Zhou XZ, Lu KP (2009). Essential role of Pin1 in the regulation of TRF1 stability and telomere maintenance. Nature cell biology.

[15] Zhou XZ, Lu KP (2001). The Pin2/TRF1-interacting protein PinX1 is a potent telomerase inhibitor. Cell.

[16] Baffa R, Santoro R, Bullrich F, Mandes B, Ishii H, Croce CM (2000). Definition and refinement of chromosome 8p regions of loss of heterozygosity in gastric cancer. Clinical cancer research : an official journal of the American Association for Cancer Research.

[17] Bova GS, MacGrogan D, Levy A, Pin SS, Bookstein R, Isaacs WB (1996). Physical mapping of chromosome 8p22 markers and their homozygous deletion in a metastatic prostate cancer. Genomics.

[18] Kishimoto Y, Shiota G, Wada K, Kitano M, Nakamoto K, Kamisaki Y, Suou T, Itoh T, Kawasaki H (1996). Frequent loss in chromosome 8p loci in liver cirrhosis accompanying hepatocellular carcinoma. Journal of cancer research and clinical oncology.

[19] Zhang B, Bai YX, Ma HH, Feng F, Jin R, Wang ZL, Lin J, Sun SP, Yang P, Wang XX, Huang PT, Huang CF, Peng Y, Chen YC, Kung HF, Huang JJ (2009). Silencing PinX1 compromises telomere length maintenance as well as tumorigenicity in telomerase-positive human cancer cells. Cancer research.

[20] Zhou XZ, Huang P, Shi R, Lee TH, Lu G, Zhang Z, Bronson R, Lu KP (2011). The telomerase inhibitor PinX1 is a major haploinsufficient tumor suppressor essential for chromosome stability in mice. The Journal of clinical investigation.

[21] Chen G, Da L, Wang H, Xu Y, Chen G, Sun C, Wang L, Zhao J, Zhang F, Feng J, Wang Y, Tiollais P, Li T, Zhao M (2011). HIV-Tat-mediated delivery of an LPTS functional fragment inhibits telomerase activity and tumorigenicity of hepatoma cells. Gastroenterology.

[22] Zhang L, Jiang Y, Zheng Y, Zeng Y, Yang Z, Huang G, Liu D, Gao M, Shen X, Wu G, Yan X, He F (2011). Selective killing of Burkitt's lymphoma cells by mBAFF-targeted delivery of PinX1. Leukemia.

[23] Zuo J, Wang DH, Zhang YJ, Liu L, Liu FL, Liu W (2013). Expression and mechanism of PinX1 and telomerase activity in the carcinogenesis of esophageal epithelial cells. Oncology reports.

[24] Ma Y, Wu L, Liu C, Xu L, Li D, Li JC (2009). The correlation of genetic instability of PINX1 gene to clinico-pathological features of gastric cancer in the Chinese population. Journal of cancer research and clinical oncology.

[25] Cai MY, Zhang B, He WP, Yang GF, Rao HL, Rao ZY, Wu QL, Guan XY, Kung HF, Zeng YX, Xie D (2010). Decreased expression of PinX1 protein is correlated with tumor development and is a new independent poor prognostic factor in ovarian carcinoma. Cancer science.

[26] Lai XF, Shen CX, Wen Z, Qian YH, Yu CS, Wang JQ, Zhong PN, Wang HL (2012). PinX1 regulation of telomerase activity and apoptosis in nasopharyngeal carcinoma cells. Journal of experimental & clinical cancer research: CR.

[27] Shi R, Zhao Z, Zhou H, Wei M, Ma WL, Zhou JY, Tan WL (2014). Reduced expression of PinX1 correlates to progressive features in patients with prostate cancer. Cancer cell international.

[28] Brooks SA, Lomax-Browne HJ, Carter TM, Kinch CE, Hall DM (2010). Molecular interactions in cancer cell metastasis. Acta histochemica.

[29] DeClerck YA, Mercurio AM, Stack MS, Chapman HA, Zutter MM, Muschel RJ, Raz A, Matrisian LM, Sloane BF, Noel A, Hendrix MJ, Coussens L, Padarathsingh M (2004). Proteases, extracellular matrix, and cancer: a workshop of the path B study section. The American journal of pathology.

[30] Giannelli G, Erriquez R, Fransvea E, Daniele A, Trerotoli P, Schittulli F, Grano M, Quaranta M, Antonaci S (2004). Proteolytic imbalance is reversed after therapeutic surgery in breast cancer patients. International journal of cancer Journal international du cancer.

[31] Karin M (2006). Nuclear factor-kappaB in cancer development and progression. Nature.

[32] Karin M, Cao Y, Greten FR, Li ZW (2002). NF-kappaB in cancer: from innocent bystander to major culprit. Nature reviews Cancer.

[33] Sato H, Takino T, Okada Y, Cao J, Shinagawa A, Yamamoto E, Seiki M (1994). A matrix metalloproteinase expressed on the surface of invasive tumour cells. Nature.

[34] Zhou XZ (2011). PinX1: a sought-after major tumor suppressor at human chromosome 8p23. Oncotarget.

[35] Lughezzani G, Jeldres C, Isbarn H, Perrotte P, Shariat SF, Sun M, Widmer H, Arjane P, Peloquin F, Pharand D, Patard JJ, Graefen M, Montorsi F, Karakiewicz PI (2009). Tumor size is a determinant of the rate of stage T1 renal cell cancer synchronous metastasis. The Journal of urology.

[36] Karakiewicz PI, Trinh QD, Bhojani N, Bensalah K, Salomon L, de la Taille A, Tostain J, Cindolo L, Altieri V, Ficarra V, Schips L, Zigeuner R, Mulders PF, Valeri A, Descotes JL, Mejean A (2007). Renal cell carcinoma with nodal metastases in the absence of distant metastatic disease: prognostic indicators of disease-specific survival. European urology.

[37] Sun M, Shariat SF, Cheng C, Ficarra V, Murai M, Oudard S, Pantuck AJ, Zigeuner R, Karakiewicz PI (2011). Prognostic factors and predictive models in renal cell carcinoma: a contemporary review. European urology.

[38] Duffy MJ (1996). The biochemistry of metastasis. Advances in clinical chemistry.

[39] Price JT, Bonovich MT, Kohn EC (1997). The biochemistry of cancer dissemination. Critical reviews in biochemistry and molecular biology.

[40] Hujanen ES, Terranova VP (1985). Migration of tumor cells to organ-derived chemoattractants. Cancer research.

[41] Fukuyama R, Ng KP, Cicek M, Kelleher C, Niculaita R, Casey G, Sizemore N (2007). Role of IKK and oscillatory NFkappaB kinetics in MMP-9 gene expression and chemoresistance to 5-fluorouracil in RKO colorectal cancer cells. Molecular carcinogenesis.

[42] Su Y, Gao L, Teng L, Wang Y, Cui J, Peng S, Fu S (2013). Id1 enhances human ovarian cancer endothelial progenitor cell angiogenesis via PI3K/Akt and NF-kappaB/MMP-2 signaling pathways. Journal of translational medicine.

[43] Prakash M, Kale S, Ghosh I, Kundu GC, Datta K (2011). Hyaluronan-binding protein 1 (HABP1/p32/gC1qR) induces melanoma cell migration and tumor growth by NF-kappa B dependent MMP-2 activation through integrin alpha(v)beta(3) interaction. Cellular signalling.

[44] Shih YW, Chien ST, Chen PS, Lee JH, Wu SH, Yin LT (2010). Alpha-mangostin suppresses phorbol 12-myristate 13-acetate-induced MMP-2/MMP-9 expressions via alphavbeta3 integrin/FAK/ERK and NF-kappaB signaling pathway in human lung adenocarcinoma A549 cells. Cell biochemistry and biophysics.

[45] Wang S, Wu X, Zhang J, Chen Y, Xu J, Xia X, He S, Qiang F, Li A, Shu Y, Roe OD, Li G, Zhou JW (2013). CHIP functions as a novel suppressor of tumour angiogenesis with prognostic significance in human gastric cancer. Gut.

[46] Wang S, Liao C, Li T, Zhao M (2004). Cloning and characterization of the promoter region of human LPTS/PinX1 gene. Biochimica et biophysica acta.

[47] Jay N, Moscicki AB (2000). Human papillomavirus infections in women with HIV disease: prevalence, risk, and management. The AIDS reader.

[48] Aravindakumar CT, Ceulemans J, De Ley M (1999). Nitric oxide induces Zn2+ release from metallothionein by destroying zinc-sulphur clusters without concomitant formation of S-nitrosothiol. The Biochemical journal.

[49] Jianfeng D, Feng J, Chaoneng J, Zhongzhou Z, Shaohua G, Qihan W, Liu W, Gang Y, Yi X, Mao Y (2003). Cloning of the correct full length cDNA of NF-kappaB-repressing factor. Molecules and cells.

[50] Nourbakhsh M, Hauser H (1999). Constitutive silencing of IFN-beta promoter is mediated by NRF (NF-kappaB-repressing factor), a nuclear inhibitor of NF-kappaB. The EMBO journal.

[51] Nourbakhsh M, Oumard A, Schwarzer M, Hauser H (2000). NRF, a nuclear inhibitor of NF-kappaB proteins silencing interferon-beta promoter. European cytokine network.

[52] Heng DY, Mackenzie MJ, Vaishampayan UN, Bjarnason GA, Knox JJ, Tan MH, Wood L, Wang Y, Kollmannsberger C, North S, Donskov F, Rini BI, Choueiri TK (2012). Primary anti-vascular endothelial growth factor (VEGF)-refractory metastatic renal cell carcinoma: clinical characteristics, risk factors, and subsequent therapy. Annals of oncology: official journal of the European Society for Medical Oncology/ESMO.

[53] Bai J, Yong HM, Chen FF, Mei PJ, Liu H, Li C, Pan ZQ, Wu YP, Zheng JN (2013). Cullin1 is a novel marker of poor prognosis and a potential therapeutic target in human breast cancer. Annals of oncology: official journal of the European Society for Medical Oncology/ESMO.

[54] Chen F, Bai J, Li W, Mei P, Liu H, Li L, Pan Z, Wu Y, Zheng J (2013). RUNX3 suppresses migration, invasion and angiogenesis of human renal cell carcinoma. PloS one.

[55] Bai J, Zhang J, Wu J, Shen L, Zeng J, Ding J, Wu Y, Gong Z, Li A, Xu S, Zhou J, Li G (2010). JWA regulates melanoma metastasis by integrin alphaVbeta3 signaling. Oncogene.

[56] Bai J, Mei PJ, Liu H, Li C, Li W, Wu YP, Yu ZQ, Zheng JN (2012). BRG1 expression is increased in human glioma and controls glioma cell proliferation, migration and invasion *in vitro*. Journal of cancer research and clinical oncology.

