# Supplementary material for: PinX1 serves as a potential prognostic indicator for clear cell renal cell carcinoma and inhibits its invasion and metastasis by suppressing MMP-2 via NF-κB-dependent transcription
Source: Oncotarget. 2015 May 27;6(25):21406–20. doi: 10.18632/oncotarget.4011 (PMC4673274; doi:10.18632/oncotarget.4011)
Supplement: Supplementary file 1 [file oncotarget-06-21406-s001.pdf]

SUPPLEMENTARY FIGURE AND TABLES

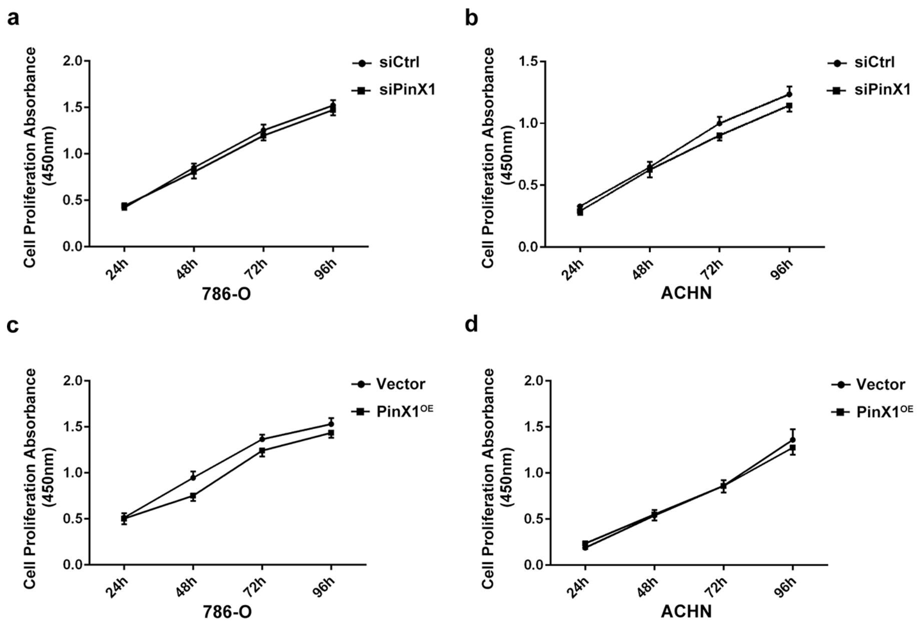

Supplementary Figure S1: PinX1 had no effect on the proliferation of ccRCC cells. a. and **b.** CCK-8 proliferation assay showed that the proliferation ability was not changed in 786-O and ACHN cells transfected with PinX1 siRNA or control siRNA. **c.** and **d.** The proliferation ability was not changed in 786-O and ACHN cells transfected with pEGFP-C3-PinX1 plasmid or vector control. All experiments were carried out in triplicate. Line chart represent means ± SD.

Supplementary Table S1: Univariate Cox proportional regression analysis on 5-year overall and disease-specific survival of 278 clear cell renal cell carcinoma patients

Variable*	Overall survival			Disease-specific survival		
	Hazard ratio	95% CI†	*P**	Hazard ratio	95% CI†	*P**
PinX1						
Low	1.000		0.003	1.000		0.002
High	0.628	0.464–0.850		0.600	0.433–0.832	
Age						
≤56 years	1.000		0.843	1.000		0.999
>56 years	1.028	0.780–1.356		1.000	0.742–1.348	
Tumor size						
≤7 cm	1.000		0.014	1.000		0.009
>7 cm	1.529	0.924–1.815		1.602	1.271–1.853	
pT status						
pT_1–pT_2	1.000		0.005	1.000		0.008
pT_3–pT_4	1.631	1.159–2.294		1.451	0.987–2.132	
pN status						
pN_0	1.000		0.003	1.000		0.005
pN_1– pN_3	2.297	1.723–2.732		2.269	2.067–2.480	
TNM stage						
I–II	1.000		0.002	1.000		0.004
III–IV	1.674	1.200–2.336		1.763	1.379–2.021	

**P* values are from Log-rank test.
†CI: confidence interval.

Supplementary Table S2: Multivariate Cox regression analysis on 5-year overall and disease-specific survival of 278 clear cell renal cell carcinoma patients

Variable*	Overall survival			Disease-specific survival		
	Hazard ratio	95% CI†	*P*	Hazard ratio	95% CI	*P*
PinX1	0.640	0.469 to 0.874	0.005	0.611	0.436 to 0.857	0.004
Age	0.980	0.731 to 1.313	0.892	0.948	0.690 to 1.303	0.742
Tumor size	1.521	1.358 to 1.802	0.021	1.480	0.964 to 1.861	0.033
TNM stage	1.509	1.053 to 2.163	0.025	1.720	1.388 to 1.952	0.025

*Coding of variables: PinX1 was coded as 1 (low), and 2 (high). Age was coded as 1 (≤56 years), and 2 (>56 years). Tumor size was coded as 1 (≤7 cm), and 2 (>7 cm). TNM stage was coded as 1 (I–II), and 2 (III–IV).
†CI: confidence interval.
